# Absence of high red blood cell velocity in the sublingual microcirculation of patients with hyerdynamic septic shock

**DOI:** 10.1186/2197-425X-3-S1-A412

**Published:** 2015-10-01

**Authors:** VS Kanoore Edul, C Ince, A Risso Vazquez, PN Rubatto, ED Valenzuela Espinoza, S Welsh, C Enrico, A Dubin

**Affiliations:** Hospital Fernández, Servicio de Terapia Intensiva, Buenos Aires, Argentina; Academic Medical Center, University of Amsterdam, Translational Physiology, Amsterdam, the Netherlands; Sanatorio Otamendi y Miroli, Servicio de Terapia Intensiva, Buenos Aires, Argentina

## Introduction

A quantitative assessment of the microcirculation in patients with septic shock previously found that microvascular flow and red blood cell (RBC) velocity were decreased^1^. This study, however, mainly included patients with normodynamic septic shock.

## Objectives

To show the presence of increased RBCV velocity in the sublingual microcirculation of patients with hyperdynamic septic shock.

## Methods

We evaluated the sublingual microcirculation of patients with hyperdynamic (n = 20) and normodynamic (n = 20) septic shock, and in healthy volunteers (n = 20). We defined hyperdynamic septic shock as a cardiac index >4.0 L/min/m^2^. Videos were acquired with a SDF-imaging device and analyzed with the AVA 3.0 software. Microvascular variables were compared with one-way ANOVA. Histograms of RBC velocities were built.

## Results

The perfused vascular density (14.7 ± 4.4, 14.1 ± 3.9, 17.4 ± 1.1 mm/mm^2^ , *P* < 0.05), the proportion of perfused vessels, (0.84 ± 0.24, 0.85 ± 0.23, 1.00 ± 0.00, *P* < 0.0001), the microvascular flow index (2.4 ± 0.7, 2.4 ± 0.7, 3.0 ± 0.0, *P* < 0.0001), and the RBC velocity (912 ± 291, 968 ± 204, 1303 ± 120 µm/s, *P* < 0.0001) were similar in hyperdynamic and normodynamic septic shock, but lower than in healthy volunteers. Both hyperdynamic and normodynamic septic shock did not have small microvessels with RBC velocity higher than the percentile 1.0 of the healthy volunteers.

## Conclusions

As occurred in normodynamic septic shock, high RBC velocity was absent in the sublingual microcirculation of patients with hyperdynamic septic shock. Moreover, the histograms of septic patients were shifted to the low range of RBC velocity.

## Grant Acknowledgment

Supported by the grant PICT-2010-00495, Agencia Nacional de Promoción Científica y Tecnológica, Argentina.

**Figure 1:**
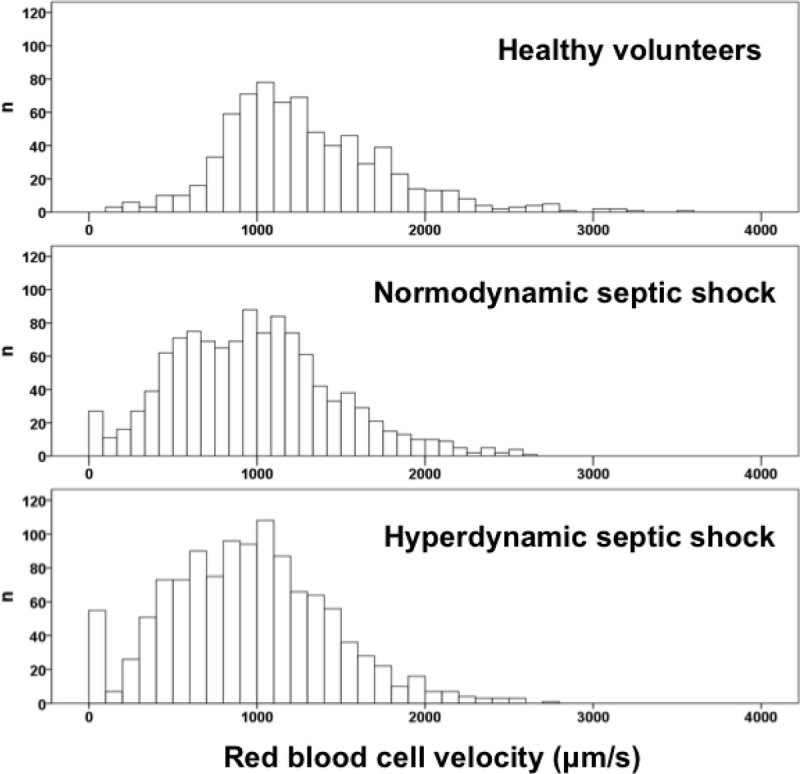
Figure 1
